# Hyaluronic acid-curcumin conjugate suppresses the fibrotic functions of myofibroblasts from contractive joint by the PTGER2 demethylation

**DOI:** 10.1093/rb/rbz016

**Published:** 2019-04-22

**Authors:** Dongjie Yu, Ze Zhuang, Jianhua Ren, Xuefeng Hu, Zhe Wang, Jieyu Zhang, Yuansen Luo, Kun Wang, Ronghan He, Yunbing Wang

**Affiliations:** 1 Department of Orthopedic Surgery, The Third Affiliated Hospital of Sun Yat-sen University, Guangzhou, China; 2 National Engineering Research Center for Biomaterials, Sichuan University, Chengdu, China

**Keywords:** curcumin, PTGER2, methylation, joint contracture

## Abstract

Joint contracture is a fibrotic complication induced by joint immobilization and trauma, which is characterized as excessive myofibroblast proliferation in joint capsule. The treatments of joint contracture are unsatisfied and patients are suffered from joint dysfunction. Our previous study has shown that curcumin can inhibit myofibroblast proliferation *in vitro*, but the major challenge is the low aqueous solubility and biological activity of curcumin. In this study, hyaluronic acid-curcumin (HA-Cur) conjugate was synthesized to suppress myofibroblasts in joint contracture. Cells were isolated from the joint capsules of joint contracture patients and induced to active myofibroblasts by transforming growth factor-β (TGF-β). The anti-fibrotic function and mechanisms of HA-Cur were investigated by immunohistochemistry, reverse transcription-quantitative polymerase chain reaction (PCR), methylation-specific PCR, western blot, transwell migration assay and proliferation assay. Results showed that 30 μM HA-Cur significantly attenuated the fibrotic functions of myofibroblast in joint contracture *in vitro* by regulating the methylation of prostaglandin E receptor 2 (PTGER2) and inhibiting TGF-β signaling. This may provide a mechanism for the treatment of joint contracture, and provide a molecular target PTGER2 for therapy during the pathogenesis of joint contracture.

## Introduction

Joint contracture is a central complication after joint surgery or longtime immobilization, which will result in irreversible joint disability [1]. Contractive joint is a common complication which affects at least 34% of patients who experienced joint surgery or immobilization [2]. However, only 36% of patients can achieve full recovery because the traditional treatments of joint contracture (rehabilitation or surgery resection of fibrotic tissue) were dissatisfactory [3–5]. The hallmark pathological changes in joint contracture are myofibroblasts (active fibroblasts) proliferation and the deposition of extracellular matrixes (ECM) in joint capsule [6]. Therefore, elucidating the mechanisms of myofibroblasts is essential to the development of joint contracture and molecules which suppress myofibroblast functions are possible treatments to prevent joint contracture.

Curcumin, a polyphenol pigment extracted from *Curcuma longa*, has been reported to exert anti-oxidant, anti-inflammatory and anti-tumor effects [7]. We previously demonstrated that curcumin can significantly suppress myofibroblast proliferation, fibrotic marker expressions as well as collagen synthesis *in vitro* [8]. Studies over the past few years have reported that curcumin could inhibit fibrotic diseases in different organs. For example, curcumin prevented transforming growth factor-β (TGF-β) inducing plasminogen activator inhibitor 1 (PAI-1) and α-smooth muscle actin (α-SMA) expressions of myofibroblasts in renal fibrosis [9]. In another study of liver fibrosis, curcumin treatment reversed liver fibrosis by demethylation of fibrotic genes, indicating that aberrant methylation was closely associated with fibrotic pathogenesis [10]. Although curcumin is potential for anti-fibrosis treatment, the major challenge is its poor aqueous solubility and low availability in biological systems [11]. Hyaluronic acid (HA), a natural polysaccharide drug which is the standard clinical intra-articular treatment of knee osteoarthritis, has a strong affinity with cell-specific markers [12]. The HA-Curcumin conjugate (HA-Cur) elevated the solubility of curcumin in water to 7.5 mg/ml, which is equivalent of 265 μM of curcumin [13]. However, it remains obscure whether curcumin can suppress the myofibroblasts from joint contracture, and, if it is, what is the exact mechanism and signaling pathway in the inhibition of myofibroblasts induced by curcumin.

Prostaglandin E2 (PGE2), a lipid mediator derived from the cyclooxygenase metabolism of arachidonic acid, potently inhibits myofibroblast functions such as cell proliferation, migration and ECM accumulation [14–19]. In some fibrotic diseases, such as idiopathic pulmonary fibrosis (IPF), the inhibition of PGE2 expression in myofibroblasts was due to the decreased expression of the prostaglandin E receptor 2 (PTGER2), the major G protein-coupled receptor of PGE2 [20, 21]. Moreover, the PTGER2 promoter contains numerous CpG dinucleotides susceptible to methylation [22, 23]. Thus, it was reported that DNA methylation is responsible for the decreased PTGER2 expression [24]. These findings strongly suggest a causal role of methylation of PTGER2 in fibrotic pathogenesis.

In the present study, we hypothesized that HA-Cur conjugate would be a solution to suppress the fibrotic functions of myofibroblasts from contractive joint. To verify this hypothesis, HA-Cur conjugate was synthesized and myofibroblasts were isolated from the posterior joint capsule. Gene, protein and tissue analyses of α-SMA, collagen type I alpha 1 (Col-I) and PTGER2 were performed by reverse transcription-quantitative polymerase chain reaction (RT-qPCR), western blot and immunohistochemistry. Myofibroblast functional experiments were conducted by transwell migration assay and myofibroblast proliferation assay. The effects of methylation of PTGER2 were determined by methylation-specific PCR (MSP) methylation inhibitor, and PTGER2 siRNA transfection, followed by myofibroblast functional tests to conform an anti-contracture mechanism.

## Materials and methods

### Cell isolation and culture

The fibroblasts were obtained from knee joint capsule of patients whose tissue histopathology is normal. All patients received informed consent. All the cells were incubated at 37°C with 5% CO_2_. The fibroblasts were cultured in DMEM (dulbecco's modified eagle medium; Keygen Biotech, Jiangsu) supplemented with 10% fetal bovine serum (FBS) (PAN SERATECH, German) and studied between passage 3–9. All the myofibroblasts in our studies were induced by TGF-β1 (Pepro Tech, USA) at concentration of 5 ng/ml for 72 h followed by 24-h serum starvation [25]. For studies on the effect of HA-Cur conjugate, we dissolved 0.85 mg HA-Cur conjugate in 1 ml of culture medium (equivalent to 30 μM of curcumin). Cells were treated for 72 h in DMEM with 10% FBS. For DNA demethylation studies, the myofibroblasts were plated at 30–50% confluence and treated with 5-aza-2′-deoxycytidine (5-aza-dC; Sigma, USA) at concentration of 5 μM for 72 h in DMEM with 10% FBS. The doses used were based on previously published reports [24, 26]. For cell transfection assays, all cells used in the study were myofibroblasts. Cells were harvested for DNA or RNA isolation. Total protein extracted from cells was subjected to western blot analysis.

### Synthesis of HA-Cur conjugate

The conjugate was synthesized as previously described [13]. In brief, 800 mg of HA (1000–1500 kDa, Yuanye Bio-Technology, Shanghai) dissolved in 1:1 V/V (H_2_O/DMSO) mixture (80 g) was added with 100 mg of 1,3-dicyclohexylcarbodiimide (DCC; Sigma, USA) and 40 mg of 4-dimethylaminopyridine (DMAP; Sigma, USA). After stirring for 1 h to activate carboxylic group of HA, the solution was mixture with 75 mg of curcumin (Sigma, USA) dissolved in 50 ml of dimethyl sulfoxide (DMSO; Sigma, USA). The mixture was stirred for about 6 h at 65°C. In order to remove unbound entities, the above solution was dialyzed against DMSO for 1 day and against deionized water (Keygen Biotech, Jiangsu) for 3 days using a dialysis membrane (MWCO: 3500 Da; West Gene, Shanghai). HA-Cur conjugate was dehydrated with dehydrant (BestBio, Shanghai) and kept at 4°C. HA-Cur conjugate was verified by ^1^HNMR measured in DMSO-d_6_ using a 300 MHz spectrometer (Bruker Avance DPX 300).

### Ultraviolet spectrophotometer assay

Twenty milligram of curcumin was accurately weighed and dissolved in 20 ml of DMSO at 25°C to prepare for the 1.0 mg/ml standard solution of curcumin. 0.04, 0.08, 0.12, 0.24, 0.48 and 0.72 ml of standard curcumin were respectively diluted to 10 ml with DMSO to prepare a series of standard solutions. Full wavelength scanning was performed between 300 and 700 nm with an Epoch ultra-micro spectrophotometer (Bio Tek, USA). The maximum absorption peak of curcumin is 440 nm. At the wavelength of 440 nm, the absorbance of the standard solutions above was measured sequentially to obtain the concentration-absorbance standard curve of the curcumin. Then, we accurately weighed 10 mg of HA-Cur conjugate and dissolved it in 10 ml of PBS. Finally, we measured its absorbance at 440 nm and calculated the content of curcumin according to standard curve. The formula is *Y* = 0.03202 × *X* + 0.05947.

### Immunohistochemistry

For immunohistochemical analysis of PTGER2 expression in human knee joint capsule, 4-μm tissue microarray sections obtained from all fibrotic and non-fibrotic tissues were paraffin-embedded and cut to construct tissue microarrays. The primary antibody anti-PTGER2 (1:100; Abcam, USA) was used for immunohistochemistry according to the manufacturer’s instructions. Slides were routinely treated for deparaffinization and hydration, and then heated in 0.01 mM citrate buffer (pH 6.0). Endogenous peroxidase activity was blocked in 3% hydrogen peroxide in methanol for 20 min at room temperature. Immunohistochemical signals were observed under a microscope (Nikon, Japan).

### Reverse transcription-quantitative polymerase chain reaction

Total RNA was isolated from cells using 1 ml Trizol (Keygen Biotech, Jiangsu) for 5 min, and then Chloroform (200 μl; Oneshine, Guangzhou) was added. After vigorous shaking for 15 s and centrifugation (Eppendorf, German) at 12 000 g for 15 min at 4°C, the aqueous phase was collected. Isopropanol (300 μl; Oneshine, Guangzhou) was mixed and maintained at 4°C for 10 min, and the mixture was centrifuged at 12 000 g for 10 min at 4°C. One milliliter of 75% ethanol (−20°C; Keygen Biotech, Jiangsu) was used to wash precipitation. After centrifugation at 7500 g for 5 min at 4°C, the RNA was dissolved in DEPC water (Keygen Biotech, Jiangsu). The qPCR was conducted on an ABI 7500-Fast (Applied Biosystems, USA) using a One Step SYBR PrimeScript RT-PCR kit II (TaKaRa, Dalian). Glyceraldehyde-3-phosphate dehydrogenase (GAPDH) served as an internal control to normalize for differences in the quantity of total RNA in each sample, and expression was calculated using the 2^−^^ΔΔCt^ method [27]. A total of three independent experiments at least were performed. The sequences of the primers used are summarized in Supplementary Table S1.

### Western blot analysis

According to the manufacturer’s instructions, proteins were extracted using a total protein extraction kit (Keygen Biotech, Jiangsu) and detected with a bicinchoninic acid (BCA) protein assay kit (Keygen Biotech, Jiangsu). Proteins were resolved by SDS-PAGE and transferred to polyvinylidene difluoride membranes. These membranes were blocked with 5% fat-free milk in PBS-Tween 20 (Solarbio, Beijing) and incubated with primary antibodies against PTGER2 (1:1000; Abcam, USA), α-SMA (1:2000; Servicebio, Wuhan), Col-I (1:2000; Servicebio, Wuhan) or β-actin (1:3000; Servicebio, Wuhan). Membranes were then incubated with the proper horseradish peroxidase (HRP)-conjugated secondary antibodies (all at 1:3000; Servicebio, Wuhan). After subsequent washing, the immunoreactive bands were developed by enhanced chemiluminescence, exposed in the dark room and analyzed for densitometry.

### Methylation-specific PCR

Genomic DNA was extracted from the cells and bisulfite modification of the DNA was performed using Methylation-Gold Kit (Tiangen Biotech, Beijing) according to the manufacturer’s instructions. The primers of PTGER2-M and PTGER2-U represent the methylated sequence and the unmethylated sequence, respectively. They were used to amplify the promoter region of the PTGER2 that incorporated a number of CpG sites and the primers were summarized in Supplementary Table S2. MSP reactions were performed using a QuantiTect SYBR Green PCR kit (QIAGEN, Shanghai) according to the manufacture’s protocol. Methylation levels (%) were calculated as the density of the M band versus the total density of ‘U + M’. All experiments were performed at least in triplicate.

### Cell proliferation assay

Cells were plated in 96-well plates (Corning, USA) at a density of 1 × 10^3^ cells per well under respective condition. The assay was determined using a Cell Counting Kit-8 kit (Keygen Biotech, Jiangsu), measuring absorbance at 450 nm by a microplate reader (Bio Tek, USA) in accordance with the manufacturer’s instructions. The formula is as follows: cell viability (%) = (*a* − *c*)/(*b* − *c*). The ‘*a*’ is absorbance of an experimental well. The ‘*b*’ is the mean absorbance of control wells without any treatment. The ‘*c*’ is the mean absorbance of wells without treatments as well as cells.

### Cell migration

Cell migration assays were performed using 8.0 μm pore transwell chambers (Corning, USA). Cells respectively treated with or without TGF-β or HA-Cur conjugate were seeded into the upper chambers of the transwells (1 × 10^5^ cells/chamber) with no FBS. DMEM containing 10% FBS as a chemoattractant was placed in the bottom chambers. After incubation for 48 h at 37°C in a 5% CO_2_ atmosphere, the cells remaining on the upper membrane were removed with cotton wool and the cells adhering to the bottom surface were fixed in 4% paraformaldehyde (Keygen Biotech, Jiangsu) and dyed with 1% crystal violet (Oneshine, Guangzhou) for 30 min. The cells that migrated through the membrane were imaged and counted using an upright metallurgical microscope (Nikon, Japan). The experiments were repeated at least in triplicate.

### Cell transfection

Cells were seeded into 24-well plates (Corning, USA) at a density of 1 × 10^5^ cells per well. Exponentially growing cells were transfected with 50 nM of PTGER2 siRNA or negative control siRNA for 48 h using a pre-designed siRNA kit (Ribobio, Guangzhou) in accordance with the manufacturer’s instructions before the treatment of HA-Cur conjugate. The siRNA was designed by Guangzhou Ribobio Co., Ltd and the target sequence is GCCTGCAACTTCAGTGTCA.

### Statistical analysis

All data were collected in triplicate for each independent preparation and expressed as the mean ± SD. The results were analyzed by analysis of Student’s *t*-test, as appropriate, using GraphPad Prism 7.0 software (GraphPad Software, USA) with *P *<* *0.05 considered to indicate a statistically significant difference.

## Results

### 30 μM HA-Cur conjugate suppressed myofibroblast functions by inducing PTGER2 up-regulation

**Figure 1:**
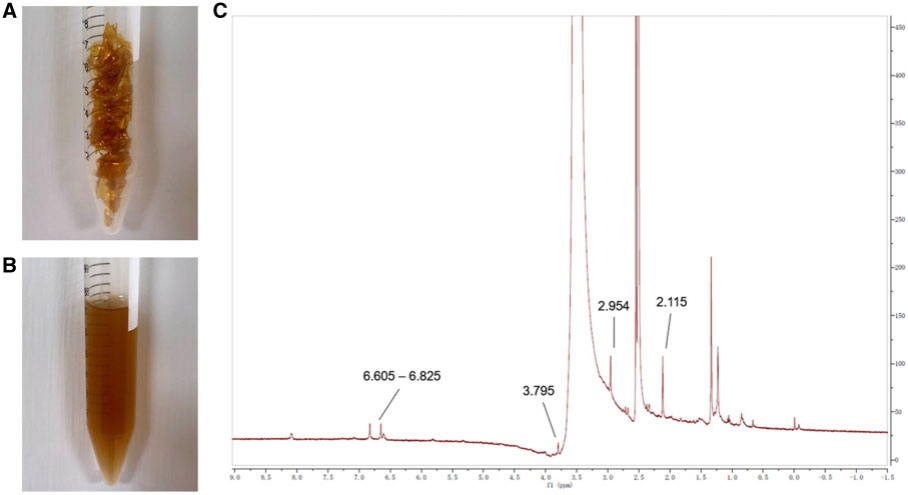
The solid state (**A**) and liquid state dissolved in PBS (**B**) of HA-Cur conjugate. (**C**) ^1^HNMR spectra of HA-Cur in DMSO-d_6_.

**Figure 2:**
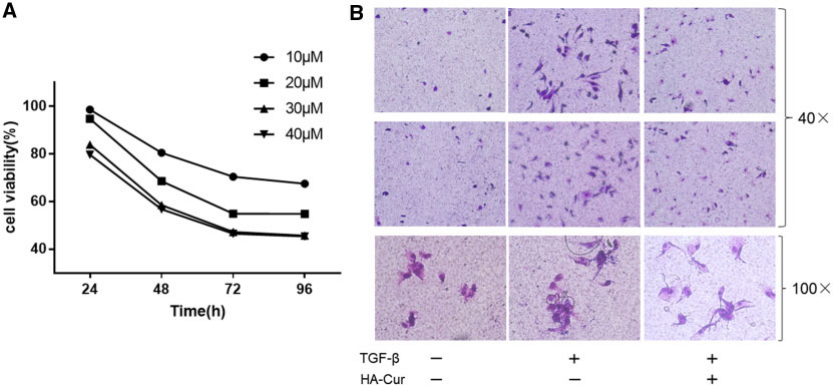
(**A**) Myofibroblast proliferation treated with different concentration of HA-Cur. (**B**) Cell migration assay of myofibroblasts treated with 30 μM of HA-Cur (*n* = 3). The above two lines (magnification, 40×) and the last line (magnification, 100×) are provided by transwell migration assay.

**Figure 3:**
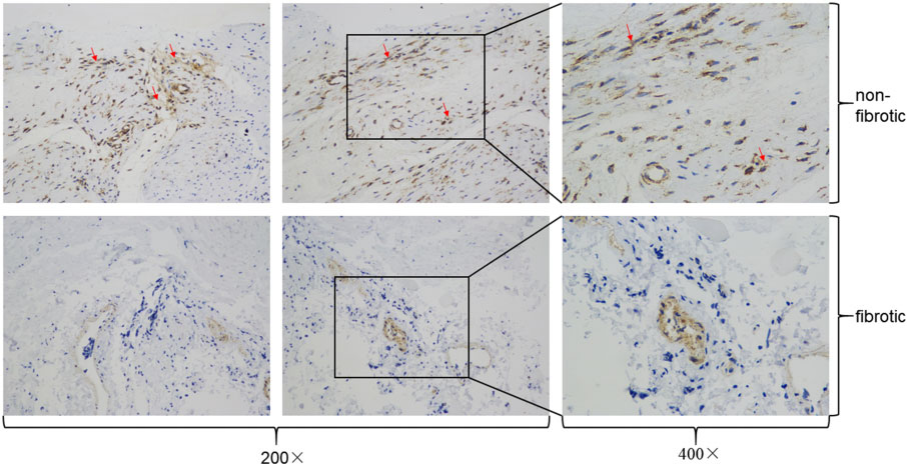
Immunohistochemistry of human knee joint capsule. The left two columns (magnification, 200×) and the right column (magnification, 400×) are from non-fibrotic and fibrotic patients, respectively.

**Figure 4:**
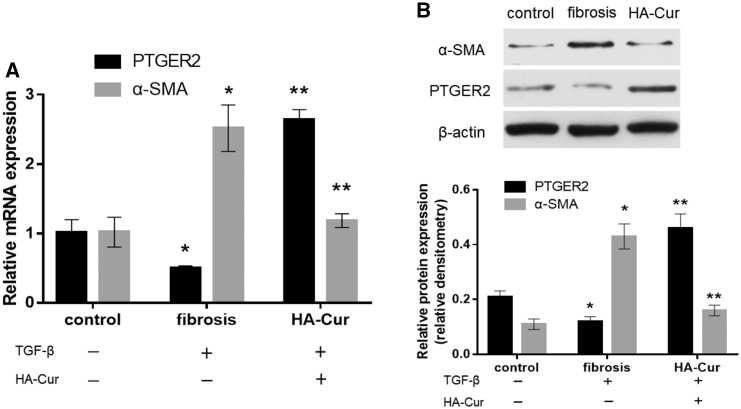
The mRNA and protein expressions of PTGER2 and α-SMA (*n* = 3). (**A**) The mRNA expressions were measured by RT-qPCR. (**B**) The protein expressions were assayed by immunoblotting, with mean densitometry relative to β-actin shown in graphs underneath the representative blot. Control group: cells were treated neither TGF-β nor HA-Cur; fibrosis group: cells treated with TGF-β; HA-Cur group: cells treated with both TGF-β and HA-Cur. *P <* *0.05 relative to the control group. **P <* *0.05 relative to fibrosis group.

The synthesis of HA-Cur conjugates was confirmed by ^1^HNMR analysis (Fig. 1A–C). A strong acetyl (-NHCOCH_3_) peak was observed at 2.115 ppm along with glucosidic H at 2.954 ppm. Peaks associated with aromatic protons of curcumin were identified at 6.605–6.825 ppm. Singlet peaks at 3.795 ppm were attributed to OCH_3_ groups in the curcumin moiety of the HA-Cur conjugate. We quantified the amount of curcumin conjugated onto the HA-Cur with ultraviolet spectrophotometer assay (Supplementary Fig. S1). The mean absorbance of HA-Cur at 440 nm was 0.4787. Therefore, according to the formula (*Y* = 0.03202 × *X* + 0.05947), the actual curcumin concentration in the HA-Cur conjugate was: (0.4787 − 0.05947)/0.03202 ≈ 13.1 μg per 1 mg of HA-Cur. In the myofibroblast proliferation and migration assays, 30 μM HA-Cur conjugate significantly suppressed cellular proliferation and migration of myofibroblasts (Fig. 2). Moreover, in the immunohistochemistry of human knee joint capsule, we found that the PTGER2 expression from joint contracture was obviously lower than non-fibrotic knee joint capsule (Fig. 3). The mRNA and protein levels of PTGER2 in myofibroblasts were elevated after 30 μM HA-Cur treatment, while the α-SMA expression level was significantly reduced (*P *<* *0.05) (Fig. 4). These results indicated that HA-Cur conjugate increased PTGER2 expression and exerted protective effects against joint contracture.

### Hypermethylation of PTGER2 was observed in myofibroblasts from contractive joint, while reduced by HA-Cur treatment

**Figure 5:**
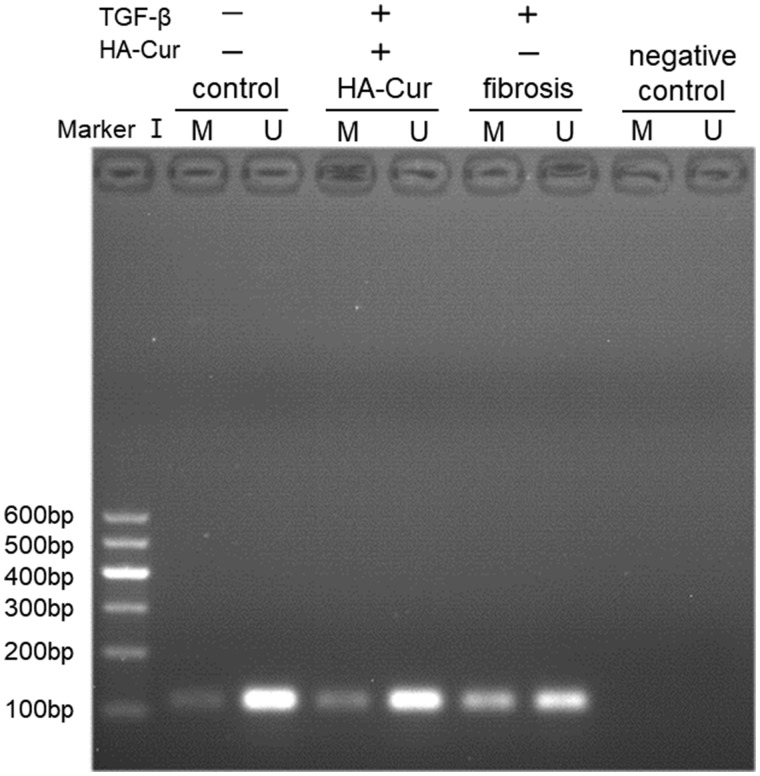
MSP Results of PTGER2 promoter methylation indicated that demethylation was observed after HA-Cur treatment (*n* = 3). U, unmethylated reactions; M, methylated reactions. The DEPC water was used as the negative control.

**Figure 6:**
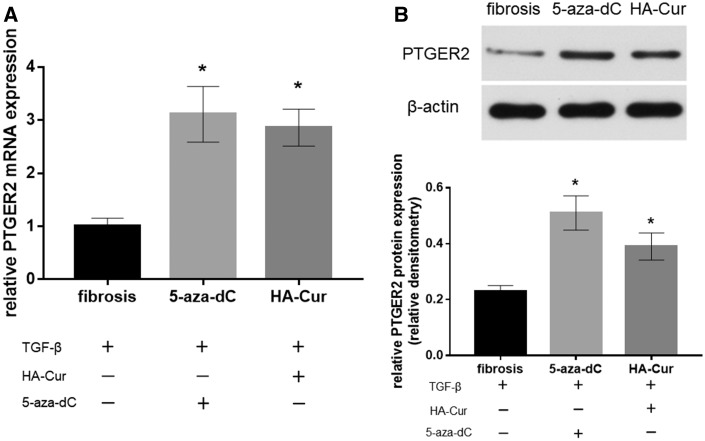
(**A**) The mRNA Expressions of myofibroblasts treated with 5-aza-dC were measured by RT-qPCR (*n* = 3). (**B**) The protein expressions were assayed by immunoblotting, with mean densitometry relative to β-actin shown in graphs underneath the representative blot. Fibrosis group: cells treated with TGF-β; 5-aza-dC group: cells treated with TGF-β and 5-aza-dC; HA-Cur group: cells treated with both TGF-β and HA-Cur. *P <* *0.05 relative to the fibrosis group.

In the MSP analysis, the hypermethylation of PTGER2 was detected in myofibroblasts from contractive joint, whereas demethylation of PTGER2 was observed after HA-Cur treatment (Fig. 5). We further treated myofibroblasts with DNA methylation inhibitor 5-aza-dC for 72 h or HA-Cur conjugate. We found that PTGER2 expression of cells treated with HA-Cur conjugate was almost at the same level compared with the cells treated with 5-aza-dC (Fig. 6).

### Regulation of PTGER2 by HA-Cur conjugate is influential on the inhibition of TGF-β signaling in myofibroblasts from contractive joint

**Figure 7:**
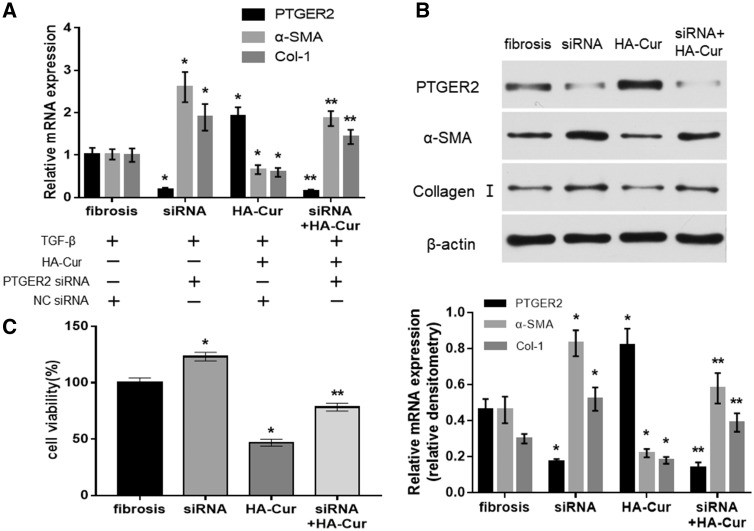
Inhibition of TGF-β signaling by HA-Cur is PTGER2-dependent in myofibroblasts (*n* = 3). (**A**) The mRNA expressions of PTGER2, α-SMA and Col-I of fibrosis group, PTGER2-siRNA group, HA-Cur group and HA-Cur + siRNA group. (**B**) The protein expressions of the above groups were assayed by immunoblotting, with mean densitometry relative to β-actin shown in graphs underneath the representative blot. (**C**) Cell proliferation in the above groups. Fibrosis group: cells treated with TGF-β and NC siRNA; siRNA group: cells treated with TGF-β and PTGER2 siRNA; HA-Cur group: cells treated with both TGF-β, HA-Cur and NC siRNA; siRNA+HA-Cur group: cells treated with. TGF-β, HA-Cur and PTGER2 siRNA. *P <* *0.05 relative to the fibrosis group. **P <* *0.05 relative to HA-Cur group.

For further studies, we investigated whether PTGER2 was influential on the inhibition of TGF-β signaling. Therefore, we employed cell proliferation and α-SMA and Col-I expressions analyses as readouts for TGF-β signaling. As shown in Fig. 7, the expressions of α-SMA and Col-I and the growth rate of myofibroblasts were obviously decreased in HA-Cur-treated myofibroblasts compared to negative control group (Fig. 7). These effects were almost blocked down by the silencing of PTGER2 with PTGER2 siRNA (Fig. 7). The results illustrated the essential role of PTGER2 in HA-Cur conjugate-mediated TGF-β signaling inhibition.

## Discussion

It has been demonstrated that myofibroblast, the activated fibroblast, is the main pathogenic cell responsible for joint contracture [6, 28]. Myofibroblasts are activated by fibrotic factors, such as TGF-β, and express fibrotic markers α-SMA and Col-I [6]. The balance of collagen deposits and ECM generation is disorganized in joint contracture [29]. In our previous study, we demonstrated that curcumin can significantly suppress myofibroblast proliferation, fibrotic markers expressions as well as collagen synthesis *in vitro* [8]. The reasons for its low bioavailability of curcumin include the low water-solubility (0.0004 mg/ml) and the rapid first-pass metabolism of the absorbed curcumin [11, 30]. Thus, a new conjugate which can improve curcumin’s solubility and bioavailability is highly desirable. In this study, HA was employed to increase aqueous solubility and availability of curcumin in the myofibroblasts inhibition. We hypothesized that HA-Cur conjugate can attenuate the fibrotic functions of myofibroblasts from contractive joint which shed a new light to employ HA-Cur for the treatment of joint contracture.

As shown in ^1^HNMR spectra, HA-Cur conjugate was indicated by specific acetyl peak (-NHCOCH_3_, 2.115 ppm), glucosidic H peak (2.954 ppm), OCH_3_ groups peak (3.795 ppm) and peaks associated with aromatic protons of curcumin were identified at 6.605–6.825 ppm (Fig. 1). This result indicated that the HA-Cur conjugate was successfully synthesized. According to Manju *et al.*, HA-Cur conjugate significantly elevated the solubility of curcumin in water to 7.5 mg/ml (equivalent to 265 μM of curcumin) and favored the stabilization of the curcumin even in alkaline media in which free curcumin rapidly degrades [13]. More importantly, the anti-fibrosis function of curcumin is not only time-dependent but also dose-dependent [10]. To explore the appropriate concentration of HA-Cur conjugate in suppressing myofibroblasts, cellular proliferation assay was performed under various curcumin concentrations (Fig. 2A). According to the proliferation result, the lowest cell viability was observed at 30 and 40 μM of HA-Cur. There was no statistically significance between these two concentrations (*P *>* *0.05). In addition, previous study showed that toxicity of curcumin occurred at doses of 20 μM in NRK49F fibroblasts [31], thus we determined a dose 30 μM on the premise of high-efficiency in suppressing myofibroblasts in our further functional experiments.

The anti-fibrosis function of HA-Cur was analyzed by cell migration assay, because the myofibroblast migration and proliferation were indispensable in joint contracture [32]. In the procession of joint contracture, the myofibroblast migration to the contractive capsule is an essential pathological process [33]. In our research, cell transwell assay was used to examine the effect of HA-Cur conjugate on myofibroblast migration (Fig. 2B). Comparing to normal fibroblasts, HA-Cur conjugate significantly reduced the migration of myofibroblasts. These data indicated that HA-Cur could not only inhibit the proliferation of myofibroblasts, but also suppress the migration of activated fibroblasts. However, although HA-Cur can suppress fibrotic formation, the anti-fibrosis mechanism of HA-Cur was still obscure.

As mentioned above, PTGER2 was reported to inhibit myofibroblast functions in some other fibrotic diseases such as IPF [20, 21]. In the immunohistochemistry of human knee joint capsule tissues, the fibrotic tissues expressed significantly less PTGER2 protein than the non-fibrotic tissues (Fig. 3). Then we further analyzed the mRNA and protein expressions of PTGER2 and α-SMA (the fibrotic marker) of cells from control group, fibrosis group and HA-Cur group. Figure 4 showed that the mRNA and protein levels of PTGER2 in myofibroblasts were elevated after 30 μM HA-Cur treatment while the α-SMA expression level was significantly reduced (*P *<* *0.05) (Fig. 4). These results indicated that HA-Cur conjugate increased PTGER2 expression and exerted protective effects against joint contracture. Hypermethylation of CpG islands in PTGER2 promoter induces a reduction in PTGER2 expression in various types of cells [24, 34, 35]. To determine whether the PTGER2 is also hypermethylated in myofibroblasts from human knee joint capsule, we performed MSP in our study. The MSP results demonstrated that the hypermethylation of PTGER2 was detected in myofibroblasts from contractive joint, whereas demethylation of PTGER2 was observed after HA-Cur treatment (Fig. 5). To further confirm whether the increased methylation of PTGER2 in myofibroblasts was responsible for decreased PTGER2 expression, we further treated myofibroblasts with DNA methylation inhibitor 5-aza-dC for 72 h or HA-Cur conjugate. Results showed that PTGER2 expression of cells treated with HA-Cur conjugate was almost at the same level compared with the cells treated with 5-aza-dC (Fig. 6). PTGER2 is a epigenetic receptor and responsible for the anti-fibrosis function. Although epigenetic alterations have been implicated in the development of many diseases [36], the role of epigenetic change, such as DNA methylation, remains relatively unclear in joint contracture. Herein, our study figured out that the increased expression of PTGER2 in myofibroblasts treated with HA-Cur conjugate was induced by PTGER2 demethylation.

**Figure 8:**
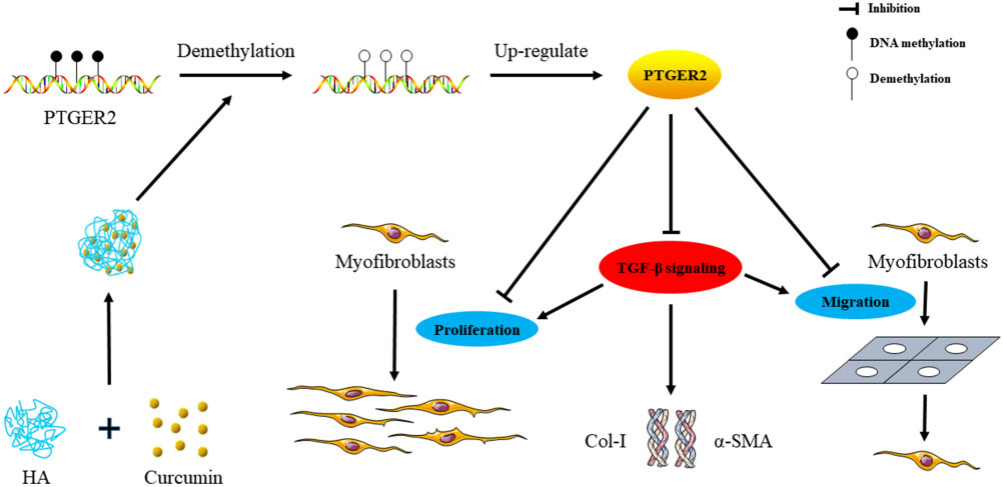
The mechanistic illustration on the effect of HA-Cur on myofibroblasts. Myofibroblasts were treated with HA-Cur conjugate in aqueous medium. HA-Cur up-regulated the PTGER2 expression, downregulated the fibrotic protein expressions and suppressed the proliferation and migration of myofibroblasts properly by PTGER2 demethylation and TGF-β signaling inhibition.

TGF-β is the master regulatory gene for epithelial-mesenchymal transitions (EMT) and is required for activation of diverse EMT biomarkers, such as α-SMA and Col-I, which are significant in fibrosis [37]. PTGER2 has been shown to regulate renal epithelial regeneration through inhibition of EMT [38]. Previous studies indicated that curcumin could reduce inflammation and inhibit cytokine expression as well as the secretion of TGF-β in fibrotic diseases [39]. In the present study, TGF-β signaling in myofibroblasts was observed to be inhibited in the treatment of HA-Cur conjugate; however, silencing PTGER2 obviated the effects of HA-Cur conjugate on TGF-β signaling, which was demonstrated by changes of Col-I and α-SMA expressions (Fig. 7). As shown in Fig. 8, our results indicated that HA-Cur conjugate alleviates myofibroblast function in joint contracture, at least partially, via the inhibition of TGF-β signaling which is PTGER2-dependent. These provided us with an insight to the mechanism of HA-Cur conjugate in joint contracture, but further researches are needed to explore underlying mechanism.

## Conclusions

Taken together, the current study indicates that HA-Cur conjugate attenuates the fibrotic functions of myofibroblasts from contractive joint *in vitro* by regulating the methylation of PTGER2 and inhibiting TGF-β signaling. This may provide a mechanism for the treatment of joint contracture, and provide a molecular target PTGER2 for therapy during the pathogenesis of joint contracture.

## Supplementary Material

rbz016_Supplementary_Data

## References

[1] Fischer U, Müller M, Strobl R et al Prevalence of functioning and disability in older patients with joint contractures: a cross-sectional study. Eur J Phys Rehabil Med 2015;51:269–79. 25192181

[2] Clavet H, Hébert PC, Fergusson D et al Joint contracture following prolonged stay in the intensive care unit. Can Med Assoc J 2008;178:691–7. 18332384 10.1503/cmaj.071056 PMC2263098

[3] Unterhauser FN, Bosch U, Zeichen J et al α-Smooth muscle actin containing contractile fibroblastic cells in human knee arthrofibrosis tissue. Arch Orthop Trauma Surg 2004;124:585–91. 15378321 10.1007/s00402-004-0742-x

[4] Pujol N, Boisrenoult P, Beaufils P. Post-traumatic knee stiffness: surgical techniques. Orthop Traumatol Surg Res 2015;101:S179–86. 25583236 10.1016/j.otsr.2014.06.026

[5] Nwachukwu BU, McFeely ED, Nasreddine A et al Arthrofibrosis after anterior cruciate ligament reconstruction in children and adolescents. J Pediatr Orthop 2011;31:811–7. 22101657 10.1097/BPO.0b013e31822e0291

[6] Abdel MP, Morrey ME, Barlow JD et al Myofibroblast cells are preferentially expressed early in a rabbit model of joint contracture. J Orthop Res 2012;30:713–9. 22057979 10.1002/jor.21588

[7] Srivastava RM, Singh S, Dubey SK et al Immunomodulatory and therapeutic activity of curcumin. Int Immunopharmacol 2011;11:331–41. 20828642 10.1016/j.intimp.2010.08.014

[8] He RH, Hu XF, Tan HC et al Surface modification of titanium with curcumin: a promising strategy to combat fibrotic encapsulation. J Mater Chem B 2015;3:2137–46. 10.1039/c4tb01616e 32262382

[9] Hu Y, Liang H, Du Y et al Curcumin inhibits transforming growth factor-beta activity via inhibition of Smad signaling in HK-2 cells. Am J Nephrol 2010;31:332–41. 20160437 10.1159/000287230

[10] Wu P, Huang R, Xiong Y et al Protective effects of curcumin against liver fibrosis through modulating DNA methylation. Chin J Nat Med 2016;14:255–64. 27114312 10.1016/S1875-5364(16)30025-5

[11] Naksuriya O, Okonogi S, Schiffelers RM et al Curcumin nanoformulations: a review of pharmaceutical properties and preclinical studies and clinical data related to cancer treatment. Biomaterials 2014;35:3365–83. 24439402 10.1016/j.biomaterials.2013.12.090

[12] Altman RD, Schemitsch E, Bedi A. Assessment of clinical practice guideline methodology for the treatment of knee osteoarthritis with intra-articular hyaluronic acid. Semin Arthritis Rheum 2015;45:132–9. 26142320 10.1016/j.semarthrit.2015.04.013

[13] Manju S, Sreenivasan K. Conjugation of curcumin onto hyaluronic acid enhances its aqueous solubility and stability. J Colloid Interface Sci 2011;359:318–25. 21492865 10.1016/j.jcis.2011.03.071

[14] Fine A, Poliks CF, Donahue LP et al The differential effect of prostaglandin E2 on transforming growth factor-beta and insulin-induced collagen formation in lung fibroblasts. J Biol Chem 1989;264:16988–91. 2676997

[15] Bitterman PB, Wewers MD, Rennard SI et al Modulation of alveolar macrophage-driven fibroblast proliferation by alternative macrophage mediators. J Clin Invest 1986;77:700–8. 3081573 10.1172/JCI112364 PMC423453

[16] Elias JA, Rossman MD, Zurier RB et al Human alveolar macrophage inhibition of lung fibroblast growth. A prostaglandin-dependent process. Am Rev Respir Dis 1985;131:94–9. 3966717 10.1164/arrd.1985.131.1.94

[17] White ES, Atrasz RG, Dickie EG et al Prostaglandin E(2) inhibits fibroblast migration by E-prostanoid 2 receptor-mediated increase in PTEN activity. Am J Respir Cell Mol Biol 2005;32:135–41. 15539459 10.1165/rcmb.2004-0126OC PMC1965457

[18] Kolodsick JE, Peters-Golden M, Larios J et al Prostaglandin E2 inhibits fibroblast to myofibroblast transition via E. prostanoid receptor 2 signaling and cyclic adenosine monophosphate elevation. Am J Respir Cell Mol Biol 2003;29:537–44. 12738687 10.1165/rcmb.2002-0243OC

[19] Huang SK, White ES, Wettlaufer SH et al Prostaglandin E(2) induces fibroblast apoptosis by modulating multiple survival pathways. FASEB J 2009;23:4317–26. 19671668 10.1096/fj.08-128801 PMC2812040

[20] Huang SK, Wettlaufer SH, Hogaboam CM et al Variable prostaglandin E2 resistance in fibroblasts from patients with usual interstitial pneumonia. Am J Respir Crit Care Med 2008;177:66–74. 17916807 10.1164/rccm.200706-963OC PMC2176116

[21] Moore BB, Ballinger MN, White ES et al Bleomycin-induced E prostanoid receptor changes alter fibroblast responses to prostaglandin E2. J Immunol 2005;174:5644–9. 15843564 10.4049/jimmunol.174.9.5644

[22] Smock SL, Pan LC, Castleberry TA et al Cloning, structural characterization, and chromosomal localization of the gene encoding the human prostaglandin E(2) receptor EP2 subtype. Gene 1999;237:393–402. 10521663 10.1016/s0378-1119(99)00323-6

[23] Regan JW, Bailey TJ, Pepperl DJ et al Cloning of a novel human prostaglandin receptor with characteristics of the pharmacologically defined EP2 subtype. Mol Pharmacol 1994;46:213–20. 8078484

[24] Huang SK, Fisher AS, Scruggs AM et al Hypermethylation of PTGER2 Confers Prostaglandin E2 Resistance in Fibrotic Fibroblasts from Humans and Mice. Am J Pathol 2010;177:2245–55. 20889571 10.2353/ajpath.2010.100446 PMC2966784

[25] Huang LS, Jiang P, Feghali-Bostwick C et al Lysocardiolipin acyltransferase regulates TGF-β mediated lung fibroblast differentiation. Free Radical Bio Med 2017;112:162–73. 28751023 10.1016/j.freeradbiomed.2017.07.023

[26] Huan C, Yang T, Liang J. Methylation-mediated BMPER expression in fibroblast activation in vitro and lung fibrosis in mice in vivo. Sci Rep 2015;5:14910. 26442443 10.1038/srep14910 PMC4595647

[27] Livak KJ, Schmittgen TD. Analysis of relative gene expression data using real-time quantitative PCR and the 2(-Delta Delta C(T)) method. Methods 2001;25:402–8. 11846609 10.1006/meth.2001.1262

[28] Wong K, Sun F, Trudel G et al Temporal gene expression profiling of the rat knee joint capsule during immobilization-induced joint contractures. BMC Musculoskelet Disord 2015;16:125. 26006773 10.1186/s12891-015-0588-0 PMC4443538

[29] Hinz B, Gabbiani G. Cell-matrix and cell-cell contacts of myofibroblasts: role in connective tissue remodeling. Thromb Haemost 2003;90:993–1002. 14652629 10.1160/TH03-05-0328

[30] Sharma RA, Gescher AJ, Steward WP. Curcumin: the story so far. Eur J Cancer 2005; 41:1955–68. 16081279 10.1016/j.ejca.2005.05.009

[31] Gaedeke J, Noble NA, Border WA. Curcumin blocks multiple sites of the TGF-beta signaling cascade in renal cells. Kidney Int 2004;66:112–20. 15200418 10.1111/j.1523-1755.2004.00713.x

[32] Satish L, Johnson S, Wang JH et al Chaperonin containing T-complex polypeptide subunit eta (CCT-eta) is a specific regulator of fibroblast motility and contractility. PLoS One 2010;5:e10063. 20442790 10.1371/journal.pone.0010063 PMC2862014

[33] Vi L, Gan BS, O’Gorman DB. The potential roles of cell migration and extra-cellular matrix interactions in Dupuytren's disease progression and recurrence. Med Hypotheses 2010;74:510–2. 19896280 10.1016/j.mehy.2009.10.009 PMC5115910

[34] Burris HH, Baccarelli AA, Motta V et al Association between length of gestation and cervical DNA methylation of PTGER2 and LINE 1-HS. Epigenetics 2014;9:1083–91. 24827772 10.4161/epi.29170 PMC4164493

[35] Tian L, Suzuki M, Nakajima T et al Clinical significance of aberrant methylation of prostaglandin E receptor 2 (PTGER2) in non-small cell lung cancer: association with prognosis, PTGER2 expression, and epidermal growth factor receptor mutation. Cancer 2008;113:1396–403. 18666211 10.1002/cncr.23694

[36] Esteller M. Epigenetic gene silencing in cancer: the DNA hypermethylome. Hum Mol Genet 2007;16:R50–9. 17613547 10.1093/hmg/ddm018

[37] Zeisberg M, Neilson EG. Biomarkers for epithelial-mesenchymal transitions. J Clin Invest 2009;119:1429–37. 19487819 10.1172/JCI36183 PMC2689132

[38] Yamamoto E, Izawa T, Juniantito V et al Involvement of endogenous prostaglandin E2 in tubular epithelial regeneration through inhibition of apoptosis and epithelial-mesenchymal transition in cisplatin-induced rat renal lesions. Histol Histopathol 2010;25:995–1007. 20552550 10.14670/HH-25.995

[39] Kliem C, Merling A, Giaisi M et al Curcumin suppresses T cell activation by blocking Ca^2+^ mobilization and nuclear factor of activated T cells (NFAT) activation. J Biol Chem 2012;287:10200–9. 22303019 10.1074/jbc.M111.318733 PMC3323045

