# Supplementary material for: Hyaluronic acid-curcumin conjugate suppresses the fibrotic functions of myofibroblasts from contractive joint by the PTGER2 demethylation
Source: Regen Biomater. 2019 Apr 22;6(5):269–77. doi: 10.1093/rb/rbz016 (PMC6783700; doi:10.1093/rb/rbz016)
Supplement: rbz016_Supplementary_Data [file rbz016_supplementary_data.docx]

**Supplementary Content**

**Hyaluronic acid-curcumin conjugate suppresses the fibrotic functions of myofibroblasts from contractive joint by the demethylation of PTGER2 promoter**

Dongjie Yu^1, †^ · Ze Zhuang^1, †^ ·Jianhua Ren^1, †^ ·Zhe Wang ^1^ · Xuefeng Hu^2^ · Jieyu Zhang^2^ · Yuansen Luo^1^ · Kun Wang^1, *^ · Ronghan He^1, *^ · Yunbing Wang^2^

^1^Department of Orthopedic Surgery, the Third Affiliated Hospital of Sun Yat-sen University, 510000, Guangzhou, China

^2^National Engineering Research Center for Biomaterials, Sichuan University, 610000, Chengdu, China

***** Corresponding author: Tel: + 86 020-85252229, Email: Kun Wang (email: wangk@mail.sysu.edu.cn) or Ronghan He (email: herh3@mail.sysu.edu.cn)

# **Table S1** List of primers sequences used in RT-qPCR.

| Gene | Sequences |
| --- | --- |
| Human PTGER2 | 5’-AGGAGACGGACCACCTCATTC-3’(Forward)  5’-GCCTAAGGATGGCAAAGACCC-3’(Reverse) |
| Human α-SMA | 5’-ACGAGACCACCTACAACAGCAT-3’(Forward)  5’-CTCGTCGTACTCCTGCTTGGT-3’(Reverse) |
| Human Col-I | 5’-GTGTTGTGCGATGACG-3’(Forward)  5’-TCGGTGGGTGACTCTG-3’(Reverse) |
| Human GAPDH | 5’-ACTTTGGTATCGTGGAAGGACTCAT-3’(Forward)  5’-GTTTTTCTAGACGGCAGGTCAGG-3’(Reverse) |

# **Table S2** List of primers sequences used in MSP.

| Gene | Sequences |
| --- | --- |
| Human PTGER2-M | 5’-GAGTATCGGATTTCGAGGAAGC-3’(Forward)  5’-AAAATCTAAACACCCGCTAAACG-3’(Reverse) |
| Human PTGER2-U | 5’-GAGTATTGGATTTTGAGGAAGTGA-3’(Forward)  5’-AAAAAATCTAAACACCCACTAAACAC-3’(Reverse) |


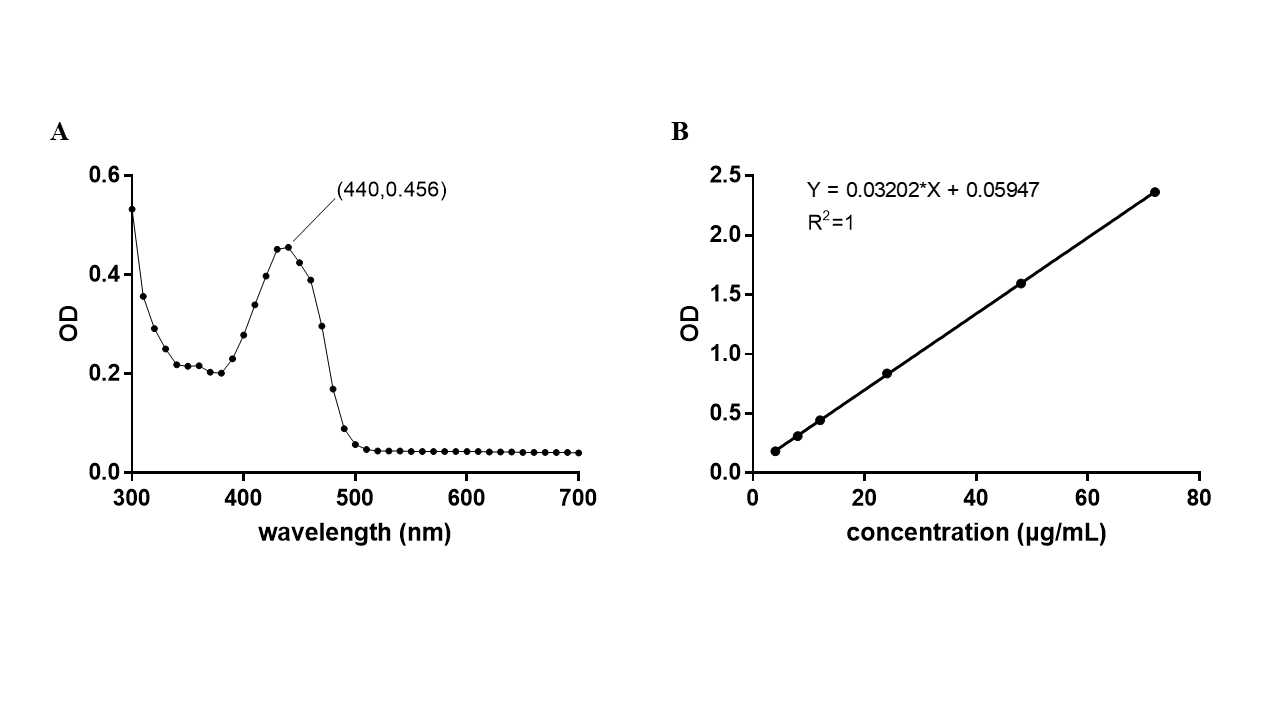


**Figure S1**. (A): Full wavelength scanning was performed between 300 nm and 700 nm and the maximum absorption peak of curcumin is 440 nm. (B) The concentration-absorbance standard curve of the curcumin.
